# Selinexor, daratumumab, and dexamethasone in patients with relapsed or refractory multiple myeloma

**DOI:** 10.1002/jha2.122

**Published:** 2020-11-08

**Authors:** Cristina Gasparetto, Suzanne Lentzsch, Gary Schiller, Natalie Callander, Sascha Tuchman, Christine Chen, Darrell White, Rami Kotb, Heather Sutherland, Michael Sebag, Muhamed Baljevic, William Bensinger, Richard LeBlanc, Chris Venner, Nizar Bahlis, Adriana Rossi, Noa Biran, Heidi Sheehan, Jean‐Richard Saint‐Martin, Dane Van Domelen, Kazuharu Kai, Jatin Shah, Sharon Shacham, Michael Kauffman, Brea Lipe

**Affiliations:** ^1^ Duke Cancer Institute School of Medicine Duke University Durham North Carolina; ^2^ Columbia University New York New York; ^3^ David Geffen School of Medicine at UCLA Los Angeles California; ^4^ University of Wisconsin Carbone Cancer Center Madison Wisconsin; ^5^ University of North Carolina Chapel Hill North Carolina; ^6^ Princess Margaret Cancer Center Toronto Ontario Canada; ^7^ Dalhousie University and QEII Health Sciences Center Halifax Nova Scotia Canada; ^8^ CancerCare Manitoba Winnipeg Manitoba; ^9^ Vancouver General Hospital Vancouver British Columbia Canada; ^10^ Royal Victoria Hospital Montreal Québec Canada; ^11^ University of Nebraska Medical Center Omaha Nebraska; ^12^ Swedish Cancer Center Seattle Washington; ^13^ CIUSSS de l'Est de l'Ile de Montréal Université de Montréal Quebec Canada; ^14^ Cross Cancer Institute Edmonton Alberta Canada; ^15^ Southern Alberta Cancer Research Institute Calgary Alberta Canada; ^16^ NYPH Weill Cornell New York New York; ^17^ Hackensack Meridian Health Hackensack University Medical Center Hackensack New Jersey; ^18^ Karyopharm Therapeutics Newton Massachusetts; ^19^ University of Rochester Medical College New York New York

**Keywords:** Selinexor, Multiple Myeloma, Daratumumab

## Abstract

We assessed the safety, efficacy, maximum tolerated dose (MTD), and the recommended phase 2 dose (RP2D) of selinexor, a first in class oral selective inhibitor of nuclear export (100 mg once weekly [QW] or 60 mg twice weekly), in combination with daratumumab (16 mg/kg per label) and dexamethasone (40 mg QW) (SDd) in patients with relapsed refractory multiple myeloma (RRMM). Thirty‐four patients (median prior therapies, 3 [range, 2‐10]) were enrolled; MM was refractory to proteasome inhibitor (PI) in 85%, immunomodulatory agent (IMiD) in 76%, both in 74%, and daratumumab in 6% of patients. Two dose‐limiting toxicities (DLTs) were reported in the selinexor 60 mg twice‐weekly cohort with no DLTs in the 100 mg QW cohort, making 100 mg QW the MTD and RP2D. Common treatment‐related adverse events included thrombocytopenia (70.6%), nausea (70.6%), fatigue (61.8%), anemia (61.8%), and neutropenia (50.0%). Overall response rate was 73% and median progression‐free survival 12.5 months in daratumumab‐naïve patients. SDd was well tolerated and its promising efficacy suggests that further study of this PI‐ and IMiD‐free regimen in RRMM patients who had at least one prior line of therapy including a PI and an IMiD but whose disease is naïve to daratumumab is warranted.

## 1 INTRODUCTION

Survival of patients with multiple myeloma (MM) has improved significantly with the introduction of new agents over the past 20 years. Specifically, bortezomib, carfilzomib, and ixazomib (proteasome inhibitors [PIs]), lenalidomide and pomalidomide (immunomodulatory agents [IMiDs]), and daratumumab (anti‐CD38 antibody) have revolutionized the MM treatment landscape [1, 2, 3, 4, 5, 6, 7, 8, 9]. Currently, combining these and other agents as triplets is standard practice based on evidence from clinical trials showing that triplets can induce deep and prolonged responses [1, 2, 4, 5, 8]. However, essentially all patients develop disease refractory to multiple agents. The survival rate of these patients with relapsed refractory MM (RRMM) remains dismal, necessitating the development of new therapies and the evaluation of new combinations [10, 11].

Exportin 1 (XPO1) is one of eight nuclear export proteins. It facilitates the transport of tumor suppressor proteins (TSPs), the glucocorticoid receptor and oncoprotein messenger RNAs (mRNAs) from the nucleus to the cytoplasm [12]. XPO1 is frequently overexpressed in MM and is associated with reduced survival and increased bone lesions [13, 14]. Selinexor is an oral selective inhibitor of nuclear export (SINE) compound that binds covalently to and inactivates XPO1, leading to the accumulation of TSPs in the nucleus, reducing translation of oncoproteins, enhancing glucocorticoid receptor signaling, and inducing cell cycle arrest, ultimately resulting in death of MM (and other malignant) cells but not in normal cells [15, 16, 17, 18, 19]. In the Phase 2b STORM study in patients with triple class (PI, IMID, and daratumumab) refractory MM, selinexor plus dexamethasone showed an overall response rate (ORR) of 26.2%, supporting the approval of selinexor in the United States [20, 21]. Selinexor continues to be evaluated in earlier lines of therapy with once‐ or twice‐weekly administration and in combination regimens. Preclinical data have shown that selinexor exhibits synergy with various anti‐MM agents [17, 22, 25]. The combination of *once weekly* (QW) selinexor with *QW* bortezomib plus low‐dose dexamethasone showed superior progression‐free survival (PFS) and ORR, a trend to reduced mortality, and significantly less peripheral neuropathy as compared with standard *twice weekly (BIW)* bortezomib in the Phase 3 BOSTON trial (NCT03110562) [26].

Daratumumab is a humanized IgG‐kappa monoclonal antibody that targets the CD38 glycoprotein on the surface of MM cells and induces specific cell cytotoxicity through antibody‐dependent binding [27]. The ORRs for daratumumab (∼29%) and selinexor (∼26%) are similar, and the associated median PFS was 3.7 months in patients with RRMM who previously received both PIs and IMiDs [3, 20]. As selinexor was shown to sensitize MM cells from newly diagnosed patients to daratumumab (Turner et al, 2017 unpublished results), we hypothesized that selinexor plus daratumumab with low‐dose dexamethasone (SDd regimen) would induce high ORR and prolonged PFS in patients with PI‐ and IMiD‐refractory MM.

The objectives of the current study were to determine the maximum tolerated dose (MTD), the recommended phase 2 dose (RP2D), and to assess the safety, tolerability, and preliminary efficacy of SDd in patients with RRMM previously treated with a PI and an IMiD.

## 2 METHODS

### 2.1 Study design and oversight

This trial is part of the larger phase 1/2b STOMP study evaluating the safety and efficacy of selinexor in combination with Food and Drug Administration‐approved therapies for RRMM (NCT02343042). Here, we report data from the dose‐escalation∖evaluation and dose‐expansion phases of varying doses of selinexor given in combination with daratumumab and low‐dose dexamethasone (SDd). In the dose‐escalation/evaluation phase, the initial dosing was not the lowest dose planned at each schedule, and subsequent doses could have been lower or higher given the safety profiles gathered at these dose levels. The primary objectives of the dose escalation phase were to determine the MTD, safety, tolerability, and identify the RP2D for the SDd regimen. The ORR and clinical benefit rate (CBR) were calculated by using data from efficacy evaluable patients. Median PFS and duration of response (DOR) were estimated by the Kaplan‐Meier method.

Patients with MM who had received ≥3 prior lines of therapy, including a PI and an IMiD, or whose MM was refractory to a PI and an IMiD, were eligible for enrollment. In the dose expansion phase, participating patients could not have received prior anti‐CD38 monoclonal antibodies. Refractory disease was defined per International Myeloma Working Group (IMWG) guidelines as lack of response while on therapy or disease progression within 2 months of completing therapy [28]. A full list of inclusion/exclusion criteria have been published previously [29]. Briefly, adults (age ≥18 years) with symptomatic, histologically confirmed, measurable MM with evidence of disease progression based on IMWG guidelines [28], an Eastern Cooperative Oncology Group (ECOG) [30] Performance Status of ≤2, and adequate hepatic, renal, and hematopoietic function were eligible for enrollment.

The study protocol was approved by the institutional review board or an independent ethics committee at each participating center and was in accordance with the Declaration of Helsinki, the International Conference on Harmonization‐Good Clinical Practice, and local laws. All patients provided written informed consent prior to enrollment. All authors reviewed the data for accuracy and collaborated in the preparation of the manuscript.

### 2.2 Treatments

In the dose‐escalation phase, patients were enrolled in two dosing cohorts using a 3 + 3 design in which patients were enrolled in blocks of three, sequentially. After three patients were enrolled in the once‐weekly selinexor dose group, the next three patients were enrolled in the twice‐weekly selinexor dose group. Patients received either selinexor 100 mg orally (PO) once weekly with dexamethasone 40 mg (intravenous [IV] or PO as single or divided doses) in 28‐day cycles or selinexor 60 mg PO BIW with dexamethasone 40 mg (IV or PO as single or divided doses) in 28‐day cycles (Table 1). For both cohorts, daratumumab 16 mg/kg IV was administered according to the package insert: weekly during weeks 1–8, then every 2 weeks during weeks 9–24, thereafter every 4 weeks ≥25 [31]. Dose escalation continued unless a dose‐limiting toxicity (DLT) was observed. DLTs were evaluated only in patients enrolled during the dose‐escalation phase over their first cycle of treatment. DLTs were defined as any of the following: (1) missing ≥25% of scheduled doses, a dose reduction, or discontinuation due to treatment‐related adverse effects (AEs) in the first cycle; (2) occurrence of grade ≥3 nausea, vomiting, dehydration, diarrhea, or fatigue lasting >3 days despite optimal supportive care medications; (3) any other grade 4 nonhematologic toxicity; (4) febrile neutropenia, grade 4 neutropenia lasting >7 days, and grade ≥3 thrombocytopenia with clinically significant bleeding, petechiae, or purpura. Electrolyte abnormalities that were reversible and asymptomatic, alopecia, alanine aminotransferase, aspartate aminotransferase, or alkaline phosphatase elevations from disease in the setting of baseline grade 2 levels were not considered to be DLTs. Infusion‐related reactions (IRRs) to daratumumab were not considered DLTs. If no DLTs occurred, the highest prespecified dose level was considered the MTD for the cohort. Patients received 5‐hydroxytryptamine‐3 (5‐HT3) antagonists (ondansetron 8 mg or equivalent, or an alternative if 5‐HT3 antagonists were not tolerated) before the first dose of selinexor and continued two to three times daily thereafter, as well as dexamethasone, oral acetaminophen, and oral diphenhydramine approximately 1 hour before the daratumumab infusion, according to the product labeling. Additional supportive care was included as part of the protocol (see Appendix for full AE management guidelines).

**TABLE 1:** Treatment schedule and dose

			Selinexor	Dexamethasone	Daratumumab
	Dose level	No. of patients	Days 1, 8, 15, 22	Weekly	Weekly for weeks 1‐8, every 2 weeks for weeks 9‐24, then every 4 weeks for weeks ≥25
Once‐weekly selinexor (QW)	1	31	100 mg orally	40 mg IV or orally	16 mg/kg IV
	Dose level	No. of patients	Days 1, 3, 8, 10, 15, 17	Weekly	Weekly for weeks 1‐8, every 2 weeks for weeks 9‐24, then every 4 weeks for weeks ≥25
Twice‐weekly selinexor (BIW)	1	3	60 mg orally	40 mg IV or orally	16 mg/kg IV

### 2.3 Study assessments

Safety was monitored throughout the study, and severity was assessed according to the National Cancer Institute Common Terminology Criteria for Adverse Events, v4.03. All patients who received at least one dose of therapy were considered evaluable for safety. Efficacy was assessed using modified IMWG guidelines [28].

### 2.4 Statistics

The sample size for the dose escalation phase of the study was based on the standard 3 + 3 dose escalation scheme. The expansion phase was designed to test the null hypothesis that the true ORR was ≤0.30 against a one‐sided alternative that the true ORR was ≥0.6 (i.e., ≥60%) and required a sample size of 25 patients. The modified intent‐to‐treat (mITT) population (patients who received ≥1 dose of study drug) was subjected for analysis, where the first 10 patients treated at the RP2D were considered the first stage of the two‐stage design. If ≤3 patients responded in Stage 1, the expansion phase would be terminated. If more than patients responded, an additional 15 patients were to be enrolled to include a total of 25 patients at the RP2D. If the total number of patients responding was ≥45%, the treatment was accepted as promising for further study. This design achieved 80% power at a 1‐sided 0.10 significance level.

## 3 RESULTS

### 3.1 Patients and Treatment

A total of 34 patients were enrolled between April 26, 2017 and February 13, 2019; three patients were enrolled in the selinexor 60‐mg twice‐weekly cohort and the remaining 31 patients were enrolled in the selinexor 100‐mg once‐weekly cohort. The median age was 68.5 years (range 44–83 years), 56% were males and the median number of prior therapies was 3 (range 2–10). All patients had received a PI and IMiD, 85% and 76% had MM refractory to a PI and an IMiD, respectively, and 74% had MM refractory to both a PI (bortezomib, carfilzomib, or ixazomib) and an IMiD (lenalidomide or pomalidomide). Seven patients had only two prior lines of therapy; of these, six patients had disease refractory to both a PI and an IMID. The remaining patient was treated with bortezomib and lenalidomide and was intolerant of both. An independent committee approved the inclusion of this patient in the study. Two patients, both enrolled into the escalation phase, had disease refractory to daratumumab. Patient demographics and disease characteristics at baseline are shown in Table 2.

**TABLE 2:** Demographics and clinical characteristics

Characteristics	All Patients (n = 34)
Median age, years (range)	68.5 (44‐83)
Age, years, No. (%)	
≤64	11 (32)
65‐74	17 (50)
≥75	6 (18)
Male, No. (%)	19 (56)
ECOG performance status, No. (%)	
0	9 (26)
1	23 (68)
2	2 (6)
Median No. of years since diagnosis (range)	5.6 (0.5‐13.7)
ISS stage at initial diagnosis, No. (%)	
I	8 (24)
II	3 (9)
III	5 (15)
Unknown	18 (53)
Genetic abnormalities at initial diagnosis or screening, n (%)	
del(13)	15 (44.1)
t(11;14)	12 (35.3)
del(17p)	10 (29.4)
t(4;14)	4 (11.8)
t(14;16)	0
Median No. of prior therapies (range)	3 (2‐10)
Prior therapies, treated: refractory; No. (%)	
Bortezomib	34 (100): 21 (62)
Carfilzomib	18 (53): 13 (38)
Lenalidomide	34 (100): 22 (65)
Pomalidomide	13 (38): 11 (32)
Daratumumab	2 (6): 2 (6)
PIs*	34 (100): 29 (85)
IMiDs^†^	34 (100): 26 (76)
PIs and IMiDs	34 (100): 25 (74)
Bortezomib and Lenalidomide	34 (100): 18 (53)
Stem cell transplantation, No. (%)	
Yes	24 (71)
No	4 (12)
Unknown	6 (18)

* PIs include bortezomib, carfilzomib, and ixazomib.

^†^ IMiDs include lenalidomide and pomalidomide.

As of the cut‐off date, six (18%) patients were still receiving treatment, 17 (50%) patients discontinued due to progressive disease, five (15%) discontinued due to AEs (one each of daratumumab IRR, hyponatremia and depression, and two patients due to fatigue), five (15%) patients withdrew consent for unknown reasons, and one (3%) patient discontinued to undergo autologous hematopoietic stem cell transplantation. The patient who underwent stem cell transplantation after SDd had received two prior lines of therapy consisting of bortezomib + lenalidomide + dexamethasone (VRd) and carfilzomib + lenalidomide + dexamethasone (KRd) without any responses to these combinations but achieved a partial response with SDd.

### 3.2 Efficacy

Response was evaluated in 32 patients during the dose‐escalation and expansion phases. Two patients were excluded for response evaluation because of early withdrawals due to (a) an IRR to daratumumab that occurred on day 1 of cycle 1, and (b) depression related to dexamethasone that was reported in week 3 of cycle 1. The ORR was 73% (22/30) in daratumumab‐naïve patients; neither patient with daratumumab refractory disease responded (Table 3). Among the 25 patients who enrolled in the expansion cohort treated with the RP2D (selinexor 100 mg and dexamethasone 40 mg weekly with daratumumab 16 mg/kg), 24 patients were evaluable excluding the patient who withdrew because of the daratumumab‐IRR. In these 24 evaluable patients, ORR was 75% (18/24), which is higher than the prespecified minimum response rate of 60% sought for the combination.

**TABLE 3:** Overall response rate

		No. of patients (%)						
Group	No. of patients*	ORR	CBR	VGPR	PR^†^	MR^‡^	SD	PD
Overall	32	22 (69)	26 (81)	11 (34)	11 (34)	4 (13)	5 (16)	1 (3)
Daratumumab‐naïve	30	22 (73)	26 (87)	11 (37)	11 (37)	4 (13)	4 (13)	–
Lenalidomide‐refractory	20	13 (65)	15 (75)	6 (30)	7 (35)	2 (10)	4 (20)	1 (5)
Bortezomib‐refractory	19	13 (68)	16 (84)	5 (26)	8 (42)	3 (16)	2 (11)	1 (5)
Pomalidomide‐refractory	10	5 (50)	8 (80)	2 (20)	3 (30)	3 (30)	1 (10)	1 (10)
Bortezomib/lenalidomide‐refractory	16	11 (69)	13 (81)	4 (25)	7 (44)	2 (13)	2 (13)	1 (6)

*Note*. Responses were investigator reported according to the International Myeloma Working Group criteria.

Abbreviations: CBR, clinical benefit rate; MR, minimal response; ORR, overall response rate; PD, progressive disease; PR, partial response; SD, stable disease; VGPR, very good partial response.

* Two patients withdrew consent prior to disease follow‐up and were, therefore, not included in the analysis of response.

^†^ Out of 11 PRs, one PR was unconfirmed.

^‡^ Out of 4 MRs, one MR was unconfirmed.

Among all response‐evaluable patients (n = 32), 11 (34%) achieved a very good PR (VGPR) and 11 (34%) achieved PRs. An additional four (13%) patients had a minimal response (MR), translating to a CBR of 87% in daratumumab‐naïve patients. Responses were rapid: all 25 (100%) patients who achieved a MR or better responded within the first cycle of treatment. Of 11 patients who achieved a VGPR, four (36%) patients reached the VGPR within the first cycle of treatment. The median follow‐up duration for this efficacy‐evaluable population (n = 32) was 12.5 months.

Two patients had MM refractory to daratumumab. One patient, who provided consent to enroll in this study immediately after progressing within the first 4 weeks of daratumumab, pomalidomide, and dexamethasone therapy, had progressed ∼1 month after the first dose of SDd; this was the only case of immediate PD. The other patient, who received daratumumab and dexamethasone 10.9 months before enrolling in this study as the last prior therapy, achieved SD with a maximal M protein reduction of 20.6%, but withdrew consent during cycle 2.

**FIGURE 1:**
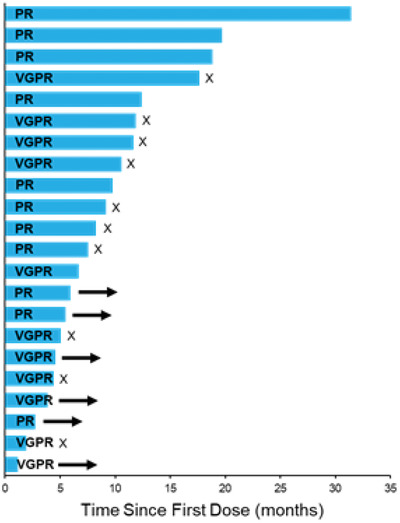
Time on study for patients with a partial response or better. Median DOR was 5.31 months (6 patients are still on treatment). Arrows indicate the study treatment is ongoing as of the date of data cutoff. X indicates disease progression

**FIGURE 2:**
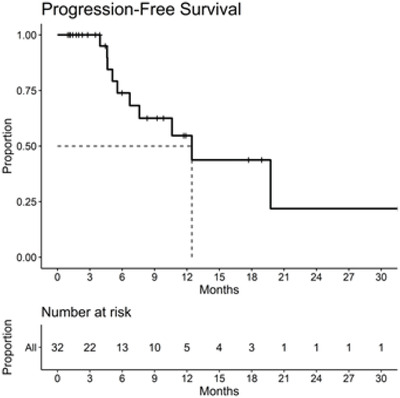
Kaplan‐Meier analysis of progression‐free survival (PFS) for all efficacy evaluable patients (n = 32). Median PFS for both all and daratumumab‐native patients was 12.5 months

**FIGURE 3:**
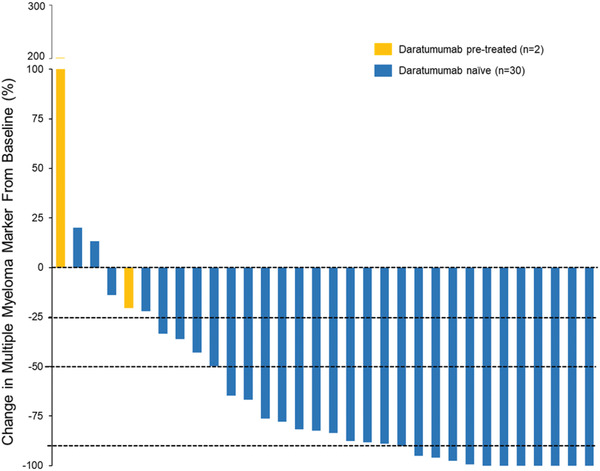
Depth of response to SDd in patients with relapse or refractory multiple myeloma (efficacy evaluable patients, n = 32). Waterfall plot depicts the best % changes in the primary myeloma marker (serum M‐protein, urine M‐protein, IgA, or serum free light chain) from baseline. The dotted line at −25%, −50%, and −90% correspond the level of reduction for a minimal response, partial response, and very good partial response, respectively

Duration of treatment is presented in Figure 1. Among responding patients, DOR was estimated as 11.4 months (95% CI: 9.7, NE). Median PFS (Figure 2) was 12.5 months in daratumumab‐naïve (n = 30) patients. Myeloma protein levels were reduced in 29 patients (91%), with 22 patients (69%) having a ≥50% reduction and 11 (34%) having a ≥90% reduction (Figure 3).

### 3.3 Safety

Two of the first three patients enrolled in the selinexor 60 mg twice‐weekly arm experienced a DLT (dose reduction in cycle 1 due to a selinexor‐related toxicity); both had reductions to selinexor 100 mg once weekly and continued on therapy to the middle of cycles 6 and 2, respectively. No DLTs were observed in the first six patients (three of which were enrolled before enrollment of any patient into the BIW group) on QW selinexor (100 mg) plus daratumumab 16 mg/kg and dexamethasone 40 mg QW; in other words, by the time two DLTs were identified in the first three patients enrolled into the Selinexor 60 mg BIW cohort, the selinexor 100 mg QW cohort already cleared DLT evaluation (out of six patients, five patients were DLT evaluable, and none of them had DLT). The safety review committee and the sponsor therefore decided not to pursue the BIW schedule anymore and determined the 100 mg QW as the MTD and the RP2D for the expansion phase.

Thirty‐four patients, nine in the escalation phase and 25 patients in expansion phase, received at least one dose of selinexor and thus were included in safety analyses (Table 4). The most common nonhematologic treatment‐related AEs were nausea (70.6%), fatigue (61.8%), dysgeusia (41.2%), diarrhea (35.3%), and anorexia (35.3%) and most were grade 1 or 2 in severity and manageable by dose modification and/or supportive care. The grade 3 or 4 nonhematological AEs that occurred in >5% of safety population were fatigue (17.6%), nausea (8.8%), and uncomplicated grade 3 hyponatremia was reported in 11.8% of patients, which was reversible (Table 4).

**TABLE 4:** Treatment‐related adverse events occurring in ≥10% patients

	No. of patients (%)			
Adverse event	Grade 1/2	Grade 3	Grade 4	Total
Hematopoietic				
Thrombocytopenia	8 (23.5)	10 (29.4)	6 (17.6)	24 (70.6)
Anemia	10 (29.4)	11 (32.4)	–	21 (61.8)
Neutropenia	8 (23.5)	9 (26.5)	–	17 (50.0)
Leukopenia	5 (14.7)	11 (32.4)	–	16 (47.1)
Lymphopenia	1 (2.9)	5 (14.7)	1 (2.9)	7 (20.6)
Gastrointestinal				
Nausea	21 (61.8)	3 (8.8)	–	24 (70.6)
Dysgeusia	14 (41.2)	–	–	14 (41.2)
Diarrhea	11 (32.4)	1 (2.9)	–	12 (35.3)
Anorexia	12 (35.3)	–	–	12 (35.3)
Vomiting	9 (26.5)	1 (2.9)	–	10 (29.4)
Constipation	10 (29.4)	–	–	10 (29.4)
Constitutional				
Fatigue	15 (44.1)	6 (17.6)	–	21 (61.8)
Weight loss	7 (20.6)	1 (2.9)	–	8 (23.5)
Dizziness	6 (17.6)	–	–	6 (17.6)
Other				
Hyponatremia	7 (20.6)	4 (11.8)	–	11 (32.4)
Insomnia	10 (29.4)	–	–	10 (29.4)
Blurred vision	10 (29.4)	–	–	10 (29.4)
Hyperglycemia	5 (14.7)	1 (2.9)	–	6 (17.6)
Dyspnea	5 (14.7)	–	–	5 (14.7)
Infusion‐related reaction	3 (8.8)	1 (2.9)	–	4 (11.8)

*Note*. Treatment‐related adverse events occurring in ≥10% of patients (n = 34). No additional grade 4 adverse events were reported other than those listed in the table. No grade 5 adverse events were reported.

Common hematologic AEs were thrombocytopenia (70.6%), anemia (61.8%), neutropenia (50.0%), and leukopenia (47.1%). Common Grade 3 or 4 hematological AEs were thrombocytopenia (47.1%), anemia (32.4%), leukopenia (32.4%), and neutropenia (26.5%). Grade 4 thrombocytopenia occurred in six patients (17.6%), one of whom (2.9%) had a bleeding event while having grade 3 thrombocytopenia. This patient was taking 2 anti‐platelet therapies, aspirin and clopidogrel, at the time. Grade 4 lymphopenia occurred in one patient (2.9%) without concurrent infection. No cases of febrile neutropenia were reported. Overall, 24 (71%) patients required selinexor dose interruptions and 22 patients (65%) required selinexor dose reductions for any cause. Among the 31 patients who were treated at the RP2D, selinexor dose interruption and reductions occurred in 21 patients (68%) and 19 patients (61%), respectively.

All but one patient received a 5‐HT3 antagonist to prevent nausea. Of these 33 patients, 23 (70%) received at least one additional antiemetic medication, and nine (27%) received two or more additional antiemetic medications; no patient withdrew due to nausea or vomiting. Eleven (32%) patients received filgrastim, four (12%) patients received the thrombopoietin receptor agonists eltrombopag or romiplostim with improvement in platelet counts, one (3%) patient received epoetin alpha, seven (21%) patients received an additional appetite stimulant (i.e., olanzapine, megesterol, and mirtazapine), and 12 (35%) patients received potassium chloride tablets.

Nine patients had at least one serious adverse event attributed to any of study drugs (selinexor, daratumumab, or dexamethasone) as follows: two rhinovirus infection (5.9% of patients), two thrombocytopenia (5.9%, no concurrent bleeding), two pneumonia (5.9%), one acute kidney injury (2.9%), one diarrhea (2.9%), one fatigue (2.9%), one hypokalemia (2.9%), one IRR (2.9%), one nausea (2.9%), and one vomiting (2.9%).

## 4 DISCUSSION

The present study was designed to determine the RP2D and assess the safety, tolerability, and preliminary efficacy of selinexor plus daratumumab and dexamethasone in patients with RRMM. Selinexor 100 mg QW was more tolerable than 60 mg BIW when combined with the standard dose of daratumumab and thus was established as the RP2D. In this heavily pretreated population, 100% had received both PIs and IMIDs and 74% had disease refractory to both drug classes. Despite this, SDd treatment among patients not refractory to daratumumab resulted in an ORR of 73%. The responses observed were relatively deep given the patient population, with VGPR rates of 37%, and durable with estimated DOR of 11.4 months and a median PFS of 12.5 months; no patient had PD as their best response.

Selinexor has shown synergistic antitumor effects in combination with PIs and IMiDs in preclinical studies and in other arms of the current study (STOMP) [17, 22, 23, 31, 32]. Given selinexor's promising activity with other active drug classes for treating MM, and the preclinical findings that combinations of CD38 engagement and XPO1 inhibition are additive or synergistic in killing MM cells (Turner et al, 2017 unpublished results), the current study was conducted to test whether selinexor's synergy with daratumumab will translate to patients with MM. The current results support the hypothesis that re‐activation of TSPs via XPO1 inhibition can enhance the activities daratumumab in heavily pretreated MM.

The safety profile of SDd was similar to that observed with selinexor along with a low rate of IRRs and cytopenias from daratumumab. Common hematological treatment‐related AEs were thrombocytopenia (70.6% in all grades, 47.1% in grade 3 or 4) with one bleeding event (2.9%), anemia (61.8%, 32.4% respectively), and neutropenia (50.0% and 26.5%, respectively; no febrile neutropenia). Another daratumumab triplet, daratumumab, pomalidomide, and dexamethasone (DPd) also demonstrates a safety profile similar to Pd, with the most common AE being neutropenia (overall 79.6%, grade 3 or 4 76.7%), followed by anemia (54.4% and 28.2%, respectively) and thrombocytopenia (41.7% in all grades, 19.4% in grade 3 or 4) [33]. As expected based on the selinexor profile [20], SDd had higher rates of thrombocytopenia, although the risk of concomitant bleeding was very low (one case, 2.9%), and nearly all cases were reversible and manageable using dose modifications along with appropriate supportive care including a thrombopoietin receptor agonist such as romiplostim [34]. Rates of grade ≥3 neutropenia on SDd were considerably lower than those on daratumumab‐IMiD combinations, and febrile neutropenia was not observed. Common nonhematological treatment‐related AEs were nausea, fatigue, dysgeusia, diarrhea, and anorexia, which were also similar to those observed with Sd [20]. More than one‐third of patients experienced these treatment‐related nonhematological AEs, most of which were grade 1 or 2, reversible and also managed or prevented with supportive care and dose modifications. For nausea and anorexia, use of prophylactic 5HT3 receptor antagonists (e.g., ondansetron) was mandated; addition of low‐dose olanzapine (or an NK1 antagonist) in cases of breakthrough nausea and/or vomiting were effective in mitigating these AEs. Moreover, low‐dose olanzapine (e.g., 2.5‐5.0 mg po qhs) is also recommended prophylactically in patients at risk of nausea or anorexia [34]. Olanzapine was also prescribed for anorexia and to prevent weight loss related to selinexor [35, 36, 37]. Five patients (15%) discontinued treatment because of AEs: one each due to daratumumab IRR, hyponatremia, and depression, and two patients due to fatigue. Overall, selinexor 100 mg once‐weekly (RP2D) was well tolerated while maintaining durable anti‐MM efficacy.

In conclusion, the QW combination of oral selinexor 100 mg, dexamethasone 40 mg, and IV daratumumab 16 mg/kg provided deep and durable responses in patients with heavily pretreated RRMM, 74% of whom had MM refractory to PIs and IMIDs. Therefore, further investigation into this combination for patients who had at least one prior line of therapy including a PI and an IMiD but whose disease is naïve to daratumumab is warranted.

## AUTHOR CONTRIBUTIONS

Conception and design: Sharon Shacham and Michael Kauffman. Provision of study material or patients: Cristina Gasparetto, Suzanne Lentzsch, Gary Schiller, Natalie Callander, Sascha Tuchman, Christine Chen, Darrell White, Rami Kotb, Heather Sutherland, Michael Sebag, Muhamed Baljevic, William Bensinger, Richard LeBlanc, Chris Venner, Nizar Bahlis, and Brea Lipe. Collection and assembly of data, data analysis and interpretation, manuscript writing, final approval of manuscript, and accountable for all aspects of the work: All authors.

## CONFLICT OF INTEREST

Cristina Gasparetto: No conflict of interest; Suzanne Lentzsch: *research funding—*Karyopharm and Sanofi; *patents, royalties, other intellectual property—*Caelum Bioscience; *stock and other ownership interests—*Caelum Bioscience, Mesoblast, Magenta, and Kadmon; *consulting or advisory role—*Caelum Bioscience, Sorrento, Janssen, and Celularity; Gary Schiller: *research funding*: AbbVie, Agios, Actinium, Ambit, AMGEN, ARIAD, Astellas, Leukemia & Lymphoma Society, BioMed Valley Discoveries, Inc., Bluebird Bio, Bristol‐Myers Squibb, Boehringer‐Ingleheim, Celator, Celgene, Cellerant, Constellation Pharmaceuticals, CTI BioPharma Corp., Forma, Cyclacel, Daiichi Sankyo, Deciphera, The California Institute for Regenerative Medicine (CIRM), Gamida Cell Ltd., GILEAD, Incyte, Janssen, Karyopharm, Kite Pharma, Inc., Mateon, MedImmune, Millennium, National Marrow Donor Program, National Institute of Health: National Cancer Institute, Novartis, Onconova, Onyx, Pfizer, PharmaMar, Sangamo, Stemline Therapeutics, Inc., National Marrow Donor Program, Tolero, Trovagene, University of California Davis, and University of California San Diego‐ UCHMC; *stock and other ownership interests—*Amgen, Bristol‐Myers Squibb, Pfizer, and Johnson and Johnson; *consulting or advisory role—*Incyte, Elevate Bio, AbbVie, ONO UK, Novartis, Evidera, Agios, AstraZeneca, National Institute of Health: National Cancer Institute, and Federal Drug Administration; *speakers' bureau—*Agios, Amgen, Astellas, Bristol‐Myers Squibb, Celgene, Sanofi‐Genzyme, Incyte, Janssen, Jazz, Kite (gilead)‐Yescarta, Pharmacyclics, and Stemline; Natalie Callander: *research funding—*Cellectar; Sascha Tuchman: *research funding—*Celgene, Karyopharm, Amgen, Janssen, and Sanofi; *consulting or advisory role—*Oncopeptides, Celgene, Karyopharm, Caelum, and Sanofi; *honoraria—*Celgene, Karyopharm, Caelum, and Sanofi; *speakers' bureau—*Celgene; Christine Chen: No conflict of interest; Darrell White: *consulting or advisory role*: Amgen, Antengene, Bristol Myers Squibb, Celgene, GlaxoSmithKline, Janssen, Karyopharm, Sanofi, and Takeda; *honoraria—*Amgen, Antengene, Bristol Myers Squibb, Celgene, GlaxoSmithKline, Janssen, Karyopharm, Sanofi, and Takeda; Rami Kotb: *research funding—*Merck and Sanofi*; stock and other ownership interests—*Karyopharm; *consulting or advisory role—*Celgene/BMS, Janssen, Amgen, Takeda, Sanofi, and Merck; Heather Sutherland: *honoraria*: Amgen, Celgene, Janssen, Takeda, Bristol Myers Squibb, and GlaxoSmith Kline; Michael Sebag: *consulting or advisory role—*Janssen and Karyopharm; Muhamed Baljevic: *consulting or advisory role—*Celgene Corporation, Cardinal Health, Putnam Associates, Gerson Lehrman Group, Inc., and AlphaSights; *honoraria—*Karyopharm Therapeutics Inc. clinical trial internal review committee member and NCCN Hematologic Malignancies Congress panelist; William Bensinger: *research funding—*BMS, Acetylon, Amgen, Janssen, Regeneron, and Sanofi; *consulting or advisory role—*Regeneron and BMS; *speakers' bureau—*Amgen, Janssen, BMS, Sanofi, and GSK; *travel, accommodations, and expenses*: Amgen, Janssen, BMS, Sanofi, and GSK; Richard LeBlanc: *consulting or advisory role—*Celgene Canada, Janssen Inc., Amgen Canada, Takeda Canada, Sanofi Canada; *speakers' bureau—*Celgene Canada, Janssen Inc., and Amgen Canada; Chris Venner: *honoraria—*Celgene, Johnson & Johnson, Amgen, Sanofi, and Takeda; Nizar Bahlis: *research funding—*received research support from Celegen and BMS; *honoraria—*Janssen, Celgene/BMS. Amgen, Takeda, Karyopharm, Sanofi, and GSK; Heidi Sheehan: employee and stockholder in Karyopharm Therapeutics; Jean‐Richard Saint‐Martin: employee and stockholder in Karyopharm Therapeutics; Dane Van Domelen: employee and stockholder in Karyopharm Therapeutics; Kazuharu Kai: employee and stockholder in Karyopharm Therapeutics; Jatin Shah: Executive Vice President, CMO, and stockholder in Karyopharm Therapeutics; Sharon Shacham: President, CSO, and stockholder in Karyopharm Therapeutics; Michael Kauffman: CEO and stockholder in Karyopharm Therapeutics; Brea Lipe: *research funding—*Amgen and Cellectar; *consulting or advisory role—*BMS, Janssen, and Abbvie.

## Supporting information

Supporting information

## Data Availability

The data that support the findings of this study are available on request from Karyopharm Therapeutics. To gain access, data requestors should submit a proposal to medicalinformation@karyopharm.com. The data are not publicly available due to privacy or ethical restrictions.
