# Supplementary material for: Selinexor, daratumumab, and dexamethasone in patients with relapsed or refractory multiple myeloma
Source: EJHaem. 2020 Nov 8;2(1):56–65. doi: 10.1002/jha2.122 (PMC9176052; doi:10.1002/jha2.122)
Supplement: Supplementary file 1 — Supporting information [file JHA2-2-56-s001.docx]

Supplemental information:

The following describes key sections from Clinical Study Protocol KCP-330-017 Ver 6.0 (STOMP) study (NCT02343042).

1. Study Population Selection

Patients who received ≥ 3 prior lines of therapy, including a PI and an IMiD, or whose MM is refractory to a PI and an IMiD. Additionally, patients must be anti-CD38 monoclonal antibody naïve in the Dose Expansion at the RP2D (Cohort 5.3- ONLY).

Inclusion Criteria

1. Written informed consent in accordance with federal, local, and institutional guidelines.
2. Age ≥ 18 years at the time of informed consent
3. Histologically confirmed diagnosis, measurable disease and evidence of disease progression of MM, as described below.
4. Symptomatic MM, based on IMWG guidelines. Patients must have measurable disease as defined by at least one of the following:

a. Serum M-protein ≥ 0.5 g/dL by serum protein electrophoresis (SPEP) or, for IgA myeloma, by quantitative IgA

b. Urinary M-protein excretion at least 200 mg/24 hours

c. Serum FLC ≥ 100 mg/L, provided that FLC ratio is abnormal

d. If SPEP is felt to be unreliable for routine M-protein measurement (e.g., for IgA MM), then quantitative Ig levels by nephelometry or turbidometry are acceptable.

1. Any non-hematological toxicities that patients had from treatments in previous clinical studies must have resolved to ≤ Grade 2 by Cycle 1 Day 1.
2. Eastern Cooperative Oncology Group (ECOG) Performance Status of ≤ 2.
3. Adequate hepatic function within 21 days prior to C1D1:

Total bilirubin of ≤ 1.5x ULN (except patients with Gilbert’s syndrome [hereditary indirect hyperbilirubinemia] who must have a total bilirubin of ≤ 3x ULN) *and* both AST *and* ALT < 2.0x ULN

1. Adequate renal function within 21 days prior to C1D1:

Estimated creatinine clearance of (calculated using the formula of Cockroft and Gault): ≥ 20 mL/min

1. Adequate hematopoietic function within 21 days prior to C1D1: total white blood cell (WBC) count ≥ 1,500/mm^3^, ANC ≥ 1000/mm^3^, Hb ≥ 8.0 g/dL, and platelet count ≥ 75,000/mm^3^. For expansion cohorts only, platelet counts > 50,000/mm^3^; for patients in whom ≥ 50% of bone marrow nucleated cells are plasma cells, platelets or ≥ 30,000/mm^3^ are acceptable for expansion cohorts. Patients receiving hematopoietic growth factor support, including erythropoietin (EPO), darbepoetin, granulocyte-colony stimulating factor (G-CSF), granulocyte macrophage-colony stimulating factor (GM-CSF), and platelet stimulators (e.g., eltrombopag or romiplostim) may continue to do so. However, patients in the escalation cohorts must be platelet transfusion independent for > 1 week in order to be enrolled in the study.
2. Female patients of childbearing potential must have a negative serum pregnancy test at Screening Female patients of childbearing potential and fertile male patients who are sexually active with a female of childbearing potential must use highly effective methods of contraception throughout the study and for 3 months following the last dose of study treatment.
3. Patients who received ≥ 3 prior lines of therapy, including a PI and an IMiD, or patients with MM refractory to both a PI and an IMiD.
4. Patients must not have received prior anti-CD38 monoclonal antibodies (Cohort 5.3 ONLY – Dose Expansion at RP2D).

Exclusion Criteria

Patients meeting any of the following exclusion criteria are not eligible to enroll in this study:

1. Smoldering MM
2. MM that does not express M-protein or FLC (i.e., non-secretory MM is excluded), and quantitative immunoglobulin levels cannot be used instead
3. Documented active systemic amyloid light chain amyloidosis
4. Active plasma cell leukemia
5. Blood (or blood product) transfusions and blood growth factors within 7 days of C1D1 (only for patients enrolling into the Expansion Phase)
6. Radiation, chemotherapy, or immunotherapy or any other anticancer therapy ≤ 2 weeks prior to C1D1, and radio-immunotherapy within 6 weeks prior to C1D1. Patients on long-term glucocorticoids during Screening do not require a washout period. Prior radiation is permitted for treatment of fractures or to prevent fractures as well as for pain management
7. Patients with history of SCC with residual paraplegia (Dose Escalation Phase only).
8. Treatment with an investigational anti-cancer therapy within 3 weeks prior to C1D1
9. Prior autologous stem cell transplantation < 1 month, or allogeneic stem cell transplantation < 3 months prior to C1D1
10. Active graft versus host disease after allogeneic stem cell transplantation
11. Life expectancy < 3 months
12. Major surgery within 4 weeks prior to C1D1
13. Active, unstable cardiovascular function:

a. Symptomatic ischemia, or

b. Uncontrolled clinically-significant conduction abnormalities (e.g., patients with ventricular tachycardia on antiarrhythmics are excluded; patients with 1st degree atrioventricular (AV) block or asymptomatic left anterior fascicular block/right bundle branch block (LAFB/RBBB) will not be excluded), or

c. Congestive heart failure (CHF) of New York Heart Association (NYHA) Class ≥ 3, or

d. Myocardial infarction (MI) within 3 months prior to C1D1, or

e. Ejection fraction (EF) < 50% at Screening

1. Uncontrolled active hypertension
2. Uncontrolled active infection requiring parenteral antibiotics, antivirals, or antifungals within one week prior to first dose
3. Known active hepatitis A, B or C
4. Known human immunodeficiency virus (HIV) infection or HIV seropositivity
5. Any active gastrointestinal dysfunction that prevents the patient from swallowing tablets or interferes with absorption of study treatment
6. Currently pregnant or breastfeeding
7. A serious psychiatric or medical condition which, in the opinion of the Investigator, could interfere with treatment
8. Hypersensitivity to any of the treatments for the Arm in which the patient is enrolled
9. Prior exposure to a SINE compound, including selinexor
10. **Treatment plan**

Selinexor once weekly cohort (Cohort 5.1)

Arm 5 SDd – Cohort 5.1 (28-day cycle), Cycles 1 and 2

| **Treatment** | **Week 1** | | | | | | | **Week 2** | | | | | | | **Week 3** | | | | | | | **Week 4** | | | | | | |
| --- | --- | --- | --- | --- | --- | --- | --- | --- | --- | --- | --- | --- | --- | --- | --- | --- | --- | --- | --- | --- | --- | --- | --- | --- | --- | --- | --- | --- |
|  | **D1** | **D2** | **D3** | **D4** | **D5** | **D6** | **D7** | **D8** | **D9** | **D10** | **D11** | **D12** | **D13** | **D14** | **D15** | **D16** | **D17** | **D18** | **D19** | **D20** | **D21** | **D22** | **D23** | **D24** | **D25** | **D26** | **D27** | **D28** |
| SEL  *QW* | X |  |  |  |  |  |  | X |  |  |  |  |  |  | X |  |  |  |  |  |  | X |  |  |  |  |  |  |
| DEX IV or PO^a^ | X | X | X |  |  |  |  | X | X | X |  |  |  |  | X | X | X |  |  |  |  | X | X | X |  |  |  |  |
| DARA | X |  |  |  |  |  |  | X |  |  |  |  |  |  | X |  |  |  |  |  |  | X |  |  |  |  |  |  |

Abbreviations: D = Study Day; DARA = daratumumab; DEX = dexamethasone; IV = intravenous; QW = once weekly; SDd = selinexor + dexamethasone + daratumumab; Sel = selinexor oral; X = dosing day.

^a^ Dexamethasone, or equivalent dose of other corticosteroid, may be given (per Investigator discretion) IV or PO and in divided doses or all at once (e.g., 20 mg IV or PO approximately 1 hr before DARA infusion and ~10 mg PO on 1st and 2nd days after infusion, or 40 mg [IV or PO] approximately 1 hr before DARA infusion [with no post-infusion day dosing]).

For all patients, the weekly dose of dexamethasone (or equivalent) must total 40 mg (or equivalent dose if using a different corticosteroid).

Dexamethasone (or equivalent) dosing must be consistent with the daratumumab labeling or standard institutional practice for recommended concomitant medications ([see the daratumumab package insert](file:///C:\Users\kkai\Downloads\KCP-330-017%20Protocol%20v6%20-%2014MAR2018%20-%20CLEAN.DOCX#daratumumabPI)).

Arm 5 SDd – Cohort 5.1 (28-day cycle), Cycles 3-6

| **Treatment** | **Week 1** | | | | | | | **Week 2** | | | | | | | **Week 3** | | | | | | | **Week 4** | | | | | | |
| --- | --- | --- | --- | --- | --- | --- | --- | --- | --- | --- | --- | --- | --- | --- | --- | --- | --- | --- | --- | --- | --- | --- | --- | --- | --- | --- | --- | --- |
|  | **D1** | **D2** | **D3** | **D4** | **D5** | **D6** | **D7** | **D8** | **D9** | **D10** | **D11** | **D12** | **D13** | **D14** | **D15** | **D16** | **D17** | **D18** | **D19** | **D20** | **D21** | **D22** | **D23** | **D24** | **D25** | **D26** | **D27** | **D28** |
| SEL  *QW* | X |  |  |  |  |  |  | X |  |  |  |  |  |  | X |  |  |  |  |  |  | X |  |  |  |  |  |  |
| DEX IV or PO^a^ | X | X | X |  |  |  |  | X |  |  |  |  |  |  | X | X | X |  |  |  |  | X^a^ |  |  |  |  |  |  |
| DARA | X |  |  |  |  |  |  |  |  |  |  |  |  |  | X |  |  |  |  |  |  |  |  |  |  |  |  |  |

Arm 5 SDd – Cohort 5.1 (28-day cycle), Cycles > 6

| **Treatment** | **Week 1** | | | | | | | **Week 2** | | | | | | | **Week 3** | | | | | | | **Week 4** | | | | | | |
| --- | --- | --- | --- | --- | --- | --- | --- | --- | --- | --- | --- | --- | --- | --- | --- | --- | --- | --- | --- | --- | --- | --- | --- | --- | --- | --- | --- | --- |
|  | **D1** | **D2** | **D3** | **D4** | **D5** | **D6** | **D7** | **D8** | **D9** | **D10** | **D11** | **D12** | **D13** | **D14** | **D15** | **D16** | **D17** | **D18** | **D19** | **D20** | **D21** | **D22** | **D23** | **D24** | **D25** | **D26** | **D27** | **D28** |
| SEL  *QW* | X |  |  |  |  |  |  | X |  |  |  |  |  |  | X |  |  |  |  |  |  | X |  |  |  |  |  |  |
| DEX IV or PO^a^ | X | X | X |  |  |  |  | X |  |  |  |  |  |  | X |  |  |  |  |  |  | X |  |  |  |  |  |  |
| DARA | X |  |  |  |  |  |  |  |  |  |  |  |  |  |  |  |  |  |  |  |  |  |  |  |  |  |  |  |

Selinexor twice weekly cohort (Cohort 5.2)

Arm 5 SDd – Cohort 5.2 (28-day cycle), Cycles 1 and 2

| **Treatment** | **Week 1** | | | | | | | **Week 2** | | | | | | | **Week 3** | | | | | | | **Week 4** | | | | | | |
| --- | --- | --- | --- | --- | --- | --- | --- | --- | --- | --- | --- | --- | --- | --- | --- | --- | --- | --- | --- | --- | --- | --- | --- | --- | --- | --- | --- | --- |
|  | **D1** | **D2** | **D3** | **D4** | **D5** | **D6** | **D7** | **D8** | **D9** | **D10** | **D11** | **D12** | **D13** | **D14** | **D15** | **D16** | **D17** | **D18** | **D19** | **D20** | **D21** | **D22** | **D23** | **D24** | **D25** | **D26** | **D27** | **D28** |
| SEL  *BIW* | X |  | X |  |  |  |  | X |  | X |  |  |  |  | X |  | X |  |  |  |  |  |  |  |  |  |  |  |
| DEX IV or PO^a^ | X | X | X |  |  |  |  | X | X | X |  |  |  |  | X | X | X |  |  |  |  | X | X | X |  |  |  |  |
| DARA | X |  |  |  |  |  |  | X |  |  |  |  |  |  | X |  |  |  |  |  |  | X |  |  |  |  |  |  |

Abbreviations: BIW = twice weekly; D = Study Day; DARA = daratumumab; DEX = dexamethasone; IV = intravenous; SDd = selinexor + dexamethasone + daratumumab; Sel = selinexor oral; X = dosing day.

^a^ Dexamethasone, or equivalent dose of other corticosteroid, may be given (per Investigator discretion) IV or PO and in divided doses or all at once (e.g., 20 mg IV or PO approximately 1 hr before DARA infusion and ~10 mg PO on 1st and 2nd days after infusion, or 40 mg [IV or PO] approximately 1 hr before DARA infusion [with no post-infusion day dosing]).

For all patients, the weekly dose of dexamethasone (or equivalent) must total 40 mg (or equivalent dose if using a different corticosteroid).

Dexamethasone (or equivalent) dosing must be consistent with the daratumumab labeling or standard institutional practice for recommended concomitant medications ([see the daratumumab package insert](file:///C:\Users\kkai\Downloads\KCP-330-017%20Protocol%20v6%20-%2014MAR2018%20-%20CLEAN.DOCX#daratumumabPI)).

Arm 5 SDd – Cohort 5.2 (28-day cycle), Cycles 3-6

| **Treatment** | **Week 1** | | | | | | | **Week 2** | | | | | | | **Week 3** | | | | | | | **Week 4** | | | | | | |
| --- | --- | --- | --- | --- | --- | --- | --- | --- | --- | --- | --- | --- | --- | --- | --- | --- | --- | --- | --- | --- | --- | --- | --- | --- | --- | --- | --- | --- |
|  | **D1** | **D2** | **D3** | **D4** | **D5** | **D6** | **D7** | **D8** | **D9** | **D10** | **D11** | **D12** | **D13** | **D14** | **D15** | **D16** | **D17** | **D18** | **D19** | **D20** | **D21** | **D22** | **D23** | **D24** | **D25** | **D26** | **D27** | **D28** |
| SEL  *BIW* | X |  | X |  |  |  |  | X |  | X |  |  |  |  | X |  | X |  |  |  |  |  |  |  |  |  |  |  |
| DEX IV or PO^a^ | X | X | X |  |  |  |  | X |  | X |  |  |  |  | X | X | X |  |  |  |  | X |  | X |  |  |  |  |
| DARA | X |  |  |  |  |  |  |  |  |  |  |  |  |  | X |  |  |  |  |  |  |  |  |  |  |  |  |  |

Arm 5 SDd – Cohort 5.2 (28-day cycle), Cycles > 6

| **Treatment** | **Week 1** | | | | | | | **Week 2** | | | | | | | **Week 3** | | | | | | | **Week 4** | | | | | | |
| --- | --- | --- | --- | --- | --- | --- | --- | --- | --- | --- | --- | --- | --- | --- | --- | --- | --- | --- | --- | --- | --- | --- | --- | --- | --- | --- | --- | --- |
|  | **D1** | **D2** | **D3** | **D4** | **D5** | **D6** | **D7** | **D8** | **D9** | **D10** | **D11** | **D12** | **D13** | **D14** | **D15** | **D16** | **D17** | **D18** | **D19** | **D20** | **D21** | **D22** | **D23** | **D24** | **D25** | **D26** | **D27** | **D28** |
| SEL  *BIW* | X |  | X |  |  |  |  | X |  | X |  |  |  |  | X |  | X |  |  |  |  |  |  |  |  |  |  |  |
| DEX IV or PO | X | X | X |  |  |  |  | X |  | X |  |  |  |  | X |  | X |  |  |  |  | X |  | X |  |  |  |  |
| DARA | X |  |  |  |  |  |  |  |  |  |  |  |  |  |  |  |  |  |  |  |  |  |  |  |  |  |  |  |

1. **Dose Modification**

Table 1 Pre-specified Selinexor Dose Modifications for AEs Related to Study Drug

| **Total Weekly Selinexor Dose** | **Selinexor Dose Schedule** |
| --- | --- |
| 120 mg | 60 mg BIW |
| 100 mg | 100 mg QW |
| 80 mg | 80 mg QW OR 40 mg BIW |
| 60 mg | 60 mg QW OR 40 mg and 20 mg on separate days |
| 40 mg | 40 mg QW OR 20 mg BIW |

Table 2 Supportive Care and Dose Adjustment Guidelines

| **Toxicity and Intensity** | **Selinexor Dose Modification** |
| --- | --- |
| **Fatigue (common)** | |
| Grade 1 or  Grade 2 lasting ≤ 7 days | Maintain dose. Rule out other causes of fatigue, particularly dehydration and anemia. If found to be anemic, consider transfusing for Hb < 8 g/dL.  Institute supportive care medications per institutional guidelines. Consistent with the National Comprehensive Cancer Network (NCCN) Clinical Practice Guidelines in Oncology (CPGO), the use of methylphenidate should be considered. Additional options are provided in the NCCN CPGO.  Patients with significant fatigue after several doses of selinexor may have an ongoing anti-tumor response. If fatigue is significant, consider assessment of tumor response as part of the patient’s evaluation. |
| Grade 2 lasting > 7 days or  Grade 3 | Interrupt selinexor dosing until resolved to Grade 1 or baseline. For first occurrence, if adequate supportive care resulted in fatigue improving to Grade 1 or baseline within 7 days, restart selinexor at current dose.  Institute supportive care medications as described above for Grade 1 or 2.  If fatigue recurs, for patients on BIW schedule, consider QW selinexor at the current dose, or for those on a QW schedule, restart selinexor at 1 dose level reduction. If fatigue abates, consider dose escalation.  Patients with significant fatigue after several doses of selinexor may have an ongoing anti-tumor response. If fatigue is significant, consider assessment of tumor response as part of the patient’s evaluation. |
| **Anorexia or Weight Loss** | |
| Grade 1 | Maintain dose. Rule out other causes of anorexia. Nutritional consultation can be helpful.  Consider instituting supportive care medications per institutional guidelines. Consistent with the NCCN CPGO, megesterol acetate 200-400 mg twice daily should be considered. Other options are provided in the NCCN CPGO. |
| Grade 2 | Rule out other causes of anorexia and order a nutritional consultation.  Institute supportive care medications as described for Grade 1 anorexia or weight loss above. For additional options, see NCCN CPGO.  Interrupt dosing with selinexor until improves to Grade 1 or baseline and weight stabilizes, then restart selinexor at current dose. May consider a 1-dose level reduction if weight loss does not improve after 1-week interruption.  Consult Medical Monitor to discuss persistent or second occurrence of weight loss after stabilization. |
| Grade 3 | Nutritional consultation and supportive care medications for anorexia per institutional guidelines should already be instituted. For additional options, see NCCN CPGO.  Interrupt dosing with selinexor until improves to Grade 1 or baseline and the weight stabilizes. Restart selinexor at 1 dose level reduction. |
| **Nausea, Acute (common)** | |
| Grade 1 or 2 (If intolerable or persistent Grade 2 not responsive to supportive care, follow guidelines for Grade 3) | Maintain dose. Rule out other causes of nausea.  Patients should be receiving 5HT3 antagonists unless contra-indicated. For those patients who cannot receive 5HT3 antagonists, consider D2 antagonists per NCCN CPGO. For additional options, such as NK1 antagonists, see NCCN CPGO.  If intolerable or persistent Grade 2, follow guidelines for Grade 3. |
| Grade 3 | Supportive care medications per institutional guidelines should already be instituted. For additional options, such as NK1 antagonists and D2 antagonists, see NCCN CPGO.  Interrupt selinexor dosing until resolved to Grade ≤ 2 or baseline. For first occurrence of Grade 3, if adequate supportive care resulted in an improvement to Grade 1 or baseline within 3 days, restart selinexor at current total dose and for patients receiving QW selinexor, consider dividing the dose (i.e., divide total weekly dose on Days 1 and 3. Note that doses on Days 1 and 3 do not need to be equal).  Otherwise (i.e., the recovery took longer than 3 days), restart selinexor at 1 dose level lower.  If nausea stabilizes for at least 4 weeks at Grade ≤ 1, may re-escalate to the original dose after consulting Medical Monitor. |
| **Hyponatremia (common)** | |
| Grade 1 (sodium levels < Normal to 130 mmol/L) | Maintain dose. Rule out other causes including drug (e.g., diuretic) effects. Be certain that reported sodium level is corrected for concurrent hyperglycemia (serum glucose > 150 mmol/L).  Treat hyponatremia per institutional guidelines including dietary review. Consider addition of salt tablets to patient’s diet. |
| Grade 3 with sodium levels 125-129 mmol/L without symptoms | Correct for hyperglycemia as outlined under Grade 1. Treat hyponatremia per institutional guidelines. If (corrected) sodium is Grade ≤ 3 and continues to be asymptomatic, then patient may continue current dosing provided that intravenous saline and/or salt tablets (1-3 times daily) are provided.  If Grade 3 is persistent or worsens or does not respond to treatment, dose interruptions may be considered after consulting with the Medical Monitor. |
| Grade 3 with sodium levels 120- 124 mmol/L with symptoms or Grade 4 (<120 mmol/L) | Treat per institutional guidelines. Delay selinexor until sodium resolved to Grade ≤ 1 (≥ 130 mmol/L) then reduce selinexor dose by 1 level. If serum sodium stabilizes to Grade ≤ 1 for at least 4 weeks, may re-escalate to previous dose of selinexor. |
| **Diarrhea (common)** | |
| Grade 1 or 2 | Initiate treatment for any Grade 1 diarrhea. Treat per institutional guidelines with anti-diarrheals and maintain dosing.  For persistent Grade 2 only that does not respond to anti-diarrheals within 3 days, interrupt selinexor dosing until resolved to Grade 1, then restart at the current dose level. |
| Grade 3 or 4 | Delay selinexor until resolved to Grade 1, then reduce selinexor dose by 1 dose level.  If diarrhea stabilizes for at least 4 weeks at Grade ≤ 1, may re-escalate to previous dose of selinexor after consulting with the Medical Monitor. |
| **Thrombocytopenia** | |
| Grade 1 or 2 | Maintain dose. |
| Grade 3 without bleeding | Consider platelet growth factors, e.g., romiplostim, which may take up to four weeks for an effect.  For patients on a QW selinexor schedule (e.g., 100 mg QW): continue selinexor without interruption at 1 dose level below and maintain weekly dosing.  For patients on a BIW selinexor schedule (e.g., 60 mg BIW): continue selinexor without interruption at 1 dose level below but at the total weekly dose scheduled as QW until further recovery to ≤ Grade 2 and then may resume the total weekly dose scheduled as BIW. For example, patients who were originally on dose level 100 mg QW and need to decrease to dose level of 80 mg total per week, should be dosed QW (i.e., 80 mg QW) until platelets recover to ≤ Grade 2 or baseline and then may resume the same total weekly dose of 80 mg total as a divided schedule of 40 mg BIW. Patients with stable platelet counts for ≥ 4 weeks following dose reduction may have their dose of selinexor increased by one dose level. |
| Grade 4 without bleeding | Strongly consider platelet growth factors and transfuse per clinical practice/institutional guidelines.  For patients on a QW selinexor schedule (e.g., 100 mg QW): delay dosing until recovery to ≤ Grade 3 or baseline and resume selinexor at 1 dose level lower.  For patients on a BIW selinexor schedule (e.g., 60 mg BIW): delay dosing until platelets recover to ≤ Grade 3 without bleeding and stable and resume selinexor at 1 dose level lower but at the total weekly dose scheduled as QW until further recovery to thrombocytopenia ≤ Grade 2 or baseline.  Once platelets recover to ≤ Grade 2 or baseline, then may resume the total weekly dose scheduled as BIW. For example, patients who were originally on dose level 100 mg QW and need to decrease to dose level of 80 mg total per week, should be dosed QW (i.e., 80 mg QW) until platelets recover to ≤ Grade 2 or baseline and then may resume the same total weekly dose of 80 mg total as a divided schedule of 40 mg BIW.  After stabilization for at least 4 weeks, a re-escalation may be considered after discussing with the Medical Monitor. |
| ≥ Grade 3 with bleeding | Delay dosing until the bleeding has stopped and the patient is clinically stable.  For patients on the QW schedule (e.g., 100 mg QW): delay dosing until recovers to ≤ Grade 3 without bleeding and stable and resume selinexor at 1 dose level below.  For patients on the BIW schedule (e.g., 60 mg BIW): delay dosing until platelets recover to ≤ Grade 3 without bleeding and stable and resume selinexor at 1 dose level below but at the total weekly dose scheduled as QW until further recovery to thrombocytopenia ≤ Grade 2 or baseline.  Once platelets recover to ≤ Grade 2 or baseline, then may resume the total weekly dose scheduled as BIW. For example, patients who were originally on dose level 100 mg QW and need to decrease to dose level of 80 mg total per week, should be dosed QW (i.e., 80 mg QW) until platelets recover to ≤ Grade 2 or baseline and then may resume the same total weekly dose of 80 mg total as a divided schedule of 40 mg BIW.  After stabilization for at least 4 weeks, a re-escalation may be considered after discussing with the Medical Monitor. |
| **Neutropenia** | |
| Grade 3 Neutropenia without fever | Maintain dosing with selinexor and institute colony stimulating factors, per institutional guidelines. If a dose reduction is desired, contact the Medical Monitor. |
| Grade 4 Neutropenia without fever | Institute colony stimulating factors, and delay dosing with selinexor until ANC returns to Grade ≤ 3. Resume dosing with selinexor at previous dose. If persistent, or for ≥ second occurrences, reduce selinexor by 1 dose level. After stabilization for at least 4 weeks, a re-escalation may be considered after discussing with the Medical Monitor. |
| Grade 3 or 4 Neutropenia with fever (febrile neutropenia) | Institute colony stimulating factors, and delay dosing with selinexor until the patient’s ANC returns to Grade ≤ 2 or baseline, fever has resolved, and patient’s condition is stable. Resume dose at previous dose level. If persistent, or for ≥ second occurrences, reduce selinexor by 1 dose level. If a dose reduction is desired, contact the Medical Monitor. |
| **Anemia** | |
| Treat per institutional guidelines including blood transfusions and/or erythropoietins. Consider transfusing for Hb < 8 g/dL. If possible, maintain selinexor dose as long as patient is clinically stable, but if dose reduction or interruption is desired, discuss with the Medical Monitor. | |
| **Other Selinexor-Related Adverse Events** | |
| Grade 1 or 2 | Maintain dose. Initiate standard supportive care per institutional guidelines. |
| Grade 3 | Delay dosing with selinexor until recovery to Grade ≤ 2 or baseline and resume selinexor at 1 dose level lower. Re-escalation can be considered after ≥ 4 weeks at the lower dose with reduction of the AE to Grade 1 or baseline. |
| Grade 4 | Delay dosing until resolved to Grade ≤ 2 or baseline, then resume at 1 dose level lower. If further dose reduction/interruption is desired, consultation with the Medical Monitor is required. |

All dose modifications should be based on the worst preceding toxicity.

Note: For combinations of Grade 1 or 2 adverse events (e.g., nausea, fatigue, anorexia) that significantly impair the patient’s quality of life, 1-2 doses of selinexor may be skipped and aggressive supportive care implemented. Selinexor may then be restarted at the original dose.

National Comprehensive Cancer Network. NCCN Clinical Practice Guidelines in Oncology (NCCN Guidelines): Fatigue, Palliative Care, and Antiemesis. Available at *http://www.nccn.org/professionals/physician_gls/f_guidelines.asp.*

Isolated values of ≥ Grade 3 alkaline phosphatase do NOT require dose interruption. Determination of liver versus bone etiology should be made, and evaluation of gamma-glutamyl transferase, 5’-nucleotidase, or other liver enzymes should be performed.

The possibility of overlapping toxicities with the non-selinexor drug (e.g., bortezomib) should be considered.

1. **Adverse event [AE] management guidelines/Supportive care – Concomitant Therapy**

**1. Required 5-HT3 Antagonists**

In order to minimize nausea, unless contraindicated, all patients should receive 5-HT3

antagonists (e.g., ondansetron 8 mg or equivalent), starting before the first dose of

selinexor and continued 2-3 times daily thereafter, as needed. Alternative treatment may

be provided if the patient does not tolerate 5-HT3 antagonists.

**2. Required Therapy for Non-Selinexor Drugs**

Arm 5 (SDd): Patients receiving daratumumab in Arm 5 should receive IV or oral

dexamethasone, oral acetaminophen, and oral diphenhydramine approximately 1 hour

before starting the daratumumab infusion, per that product’s labeling.

Arm 6 (SKd): Patients receiving carfilzomib in Arm 6 should receive IV or oral

dexamethasone 30 minutes to 4 hours before each carfilzomib dose in Cycle 1, then as

needed to help prevent infusion reactions, per that product’s labeling.

**3. Supportive Care**

Supportive measures for optimal medical care should be provided to patients during

participation in this study. Based on preliminary safety results in 2,103 patients treated

with selinexor as of 31 May 2017, the main side effects have been primarily related to

anorexia with poor caloric and fluid intake, fatigue, and nausea. Thrombocytopenia also

occurs, although it is rarely associated with bleeding. Required 5-HT3 prophylaxis,

supportive care including additional anti-nausea/anti-emetic therapy, acid suppression

(proton-pump inhibitors [PPI] and/or H2-blockers) and other treatments may be

administered as follows:

• Appetite stimulants: megesterol acetate at a dose of 80-400 mg daily.

• Centrally-acting agents: per *National Comprehensive Cancer Network® [NCCN]*

*Clinical Practice Guidelines*®.

• Neurokinin-1 receptor (NK1R) antagonist: Aprepitant or equivalent should be

considered and will be covered for selected patients who have severe nausea and

vomiting.

Supportive care guidelines for managing AEs are provided in Table 6.

**3.1. Infection**

Appropriate broad-spectrum IV antibiotics and antifungal agents should be started

immediately in patients who develop fever or other signs of systemic infection. Selinexor

should be suspended in any patient with Grade 4 infection or clinical sepsis (in the

absence of documented infection) until the condition is stabilized. Selinexor can then be

re-started at the same dose. See also Table 6*.*

**3.2. Glucocorticoid Side Effects**

The management of common glucocorticoid side effects is well documented. Aggressive

use of PPIs, anti-hypertensives, glucose-lowering drugs and other agents is strongly

encouraged in order to maintain the use of dexamethasone in combination with selinexor

in this study. Patients with documented osteopenia or osteoporosis should continue to take

dexamethasone with selinexor as indicated in the study. Standard precautions such as use

of bisphosphonates should be instituted unless contraindicated.

1. Multiple Myeloma Disease Assessments

Patient response will be assessed by the procedures summarized in Table 3 and graded according to IMWG (Table 4).

Table 3 . Multiple Myeloma Disease-Specific Assessments

Patients will have their disease assessed by the following procedures per modified IMWG (see Table 4). Assessments on dosing days should be performed pre-dose.

| **Procedure** | **Notes** |
| --- | --- |
| SPEP with M-spike quantification, and serum protein immunofixation | Per modified IMWG |
| UPEP (24-hour urine for total protein) with M-spike quantification and urine protein immunofixation | Per modified IMWG |
| Serum FLC | Per modified IMWG |
| Quantitative immunoglobulin (Ig) levels | If SPEP is felt to be unreliable for routine M-protein measurement, then quantitative Ig levels by nephelometry or turbidometry are acceptable.  For IgA myeloma, by quantitative IgA. |
| β_2_-microglobulin | For MM staging, not for assessing response |
| Skeletal survey | A skeletal survey (using X-rays and/or other clinically appropriate imaging [MRI, whole body CT, or PET/CT]) will be performed during Screening and as clinically indicated, per Investigator’s discretion, during the study.  If X-rays are used, they should include a lateral radiograph of skull, anterioposterior and lateral views of the spine, and anterioposterior views of the pelvis, ribs, femora, and humeri.  If lytic bone lesions or plasmacytomas are observed at Screening, their location and measurement should be recorded in the CRF. They should be re-assessed during the study, as clinically appropriate (per Investigator’s discretion) using the same imaging modality that was used at Screening. |
| Plasmacytoma | If plasmacytomas are detected at baseline by physical examination or imaging (MRI, ultrasound, whole body CT, or PET/CT), they should be measured and recorded.  Plasmacytomas that are measurable by physical exam must be assessed during the physical exam on Day 1 of each cycle.  Plasmacytomas that are seen by imaging during Screening should be re-assessed during the study as clinically appropriate (per Investigator discretion) using the same imaging modality that was used at Screening. |
| Bone marrow aspirate | The bone marrow aspirate obtained at Screening will be used for fluorescence in situ hybridization (FISH) analysis to confirm diagnosis and classify MM sub-type, and A bone marrow aspirate and/or core biopsy is/are required when there is negative immunofixation of serum and urine, and disappearance of any soft tissue plasmacytomas. |
| Bone marrow core biopsy | Investigators may optionally perform two optional bone marrow core biopsies, *in addition* to the required bone marrow aspirates. If sufficient sample is available, Karyopharm requests that one portion of each biopsy should be fixed in 10% formalin and another portion should be fresh frozen. An archival sample taken within 30 days prior to C1D1 may be used in lieu of the pre-treatment sample. The post-treatment sample should be obtained after completing one full cycle on C2D1 (+ 5 days) only from patients for whom a pre-treatment sample is also available. These samples will be used for exploratory correlative PDn studies. Patients must positively consent to these procedures on the ICF. These optional core biopsies may be stored for up to 15 years for possible future correlative studies for genomic biomarkers. |

**Table 4. International Myeloma Working Group Response Criteria, Myeloma**

*International Myeloma Working Group Response Criteria, Myeloma (*Palumbo 2014*)*

| Response Subcategory | Response Criteria |
| --- | --- |
| Complete response (CR) | Negative immunofixation of serum and urine, disappearance of any soft tissue plasmacytomas, and < 5% plasma cells in bone marrow; in patients for whom only measurable disease is by serum FLC level, normal FLC ratio of 0.26 to 1.65 in addition to CR criteria is required; two consecutive assessments are needed |
| Stringent complete response (sCR) | CR as defined above plus normal FLC ratio and absence of clonal plasma cells by immunohistochemistry or 2-to-4-color flow cytometry; two consecutive assessments of laboratory parameters are needed |
| Immunophenotypic CR | sCR as defined plus absence of phenotypically aberrant plasma cells (clonal) in bone marrow with minimum of 1 million total bone marrow cells analyzed by multiparametric flow cytometry (with > 4 colors) |
| Molecular CR | CR as defined plus negative allele-specific oligonucleotide polymerase chain reaction (sensitivity 10^-5^) |
| Very good partial response (VGPR) | Serum and urine M-component detectable by immunofixation but not on electrophoresis or ≥ 90% or greater reduction in serum M‑component plus urine M-component < 100 mg per 24 hr; in patients for whom only measurable disease is by serum FLC level, > 90% decrease in difference between involved and uninvolved FLC levels, in addition to VGPR criteria is required; two consecutive assessments are needed |
| Partial response (PR) | ≥ 50% reduction of serum M‑protein and reduction in 24-hr urinary M‑protein by ≥ 90% or to < 200 mg/24 hr.  If serum and urine M‑protein are not measurable, a ≥ 50% decrease in the difference between involved and uninvolved FLC levels is required in place of the M‑protein criteria.  If serum and urine M‑protein and serum free light chain (FLC) assay are not measurable, ≥ 50% reduction in bone marrow plasma cells is required in place of M‑protein, provided baseline percentage was ≥ 30%.  In addition to the above criteria, if present at baseline, ≥ 50% reduction in the size of soft tissue plasmacytomas is required.  Two consecutive assessments are needed; no known evidence of progressive or new bone lesions if radiographic studies were performed. |
| Minimal response (MR) in patients with RR myeloma only | ≥ 25% but < 49% reduction of serum M‑protein and reduction in 24‑hr urine M‑protein by 50–89%.  In addition to the above criteria, if present at baseline, 25–49% reduction in the size of soft tissue plasmacytomas is also required.  No increase in size or number of lytic bone lesions (development of compression fracture does not exclude response). |
| Stable disease (SD) | Not meeting criteria for CR, VGPR, PR, or PD; no known evidence of progressive or new bone lesions if radiographic studies were performed |
| Progressive disease (PD) | Increase of 25% from lowest response value in any of the following:   - Serum M-component with absolute increase ≥ 0.5 g/dL; serum M‑component increases of ≥ 1 g/dL are sufficient to define relapse if starting M-component is ≥ 5 g/dL and/or; - Urine M-component (absolute increase must be ≥ 200 mg/24 hr) and/or; - Only in patients without measurable serum and urine M-protein levels: the difference between involved and uninvolved FLC levels (absolute increase must be > 10 mg/dL). - Only in patients without measurable serum and urine M‑protein levels and without measurable disease by FLC level: bone marrow plasma cell percentage (absolute % must be ≥ 10%). - Development of new or definite increase in the size of existing bone lesions or soft tissue plasmacytomas. - Development of hypercalcemia that can be attributed solely to the plasma cell proliferative disorder. - Two consecutive assessments before new therapy are needed. |

Source: Palumbo A, Rajkumar SV, San Miguel JF, et al. International Myeloma Working Group consensus statement for the management, treatment, and supportive care of patients with myeloma not eligible for standard autologous stem-cell transplantation. *J Clin Oncol.* 2014;32:587-600.

Contents adapted from:

- Durie BG, Harousseau JL, Miguel JS, et al. International uniform response criteria for multiple myeloma. *Leukemia*. 2006;20:1467-1473.
- Kyle RA and Rajkumar SV. Criteria for diagnosis, staging, risk stratification and response assessment of multiple myeloma. *Leukemia*. 2009;23(1):3-9.
- Rajkumar SV, Larson D, Kyle RA: Diagnosis of smoldering multiple myeloma. N Engl J Med. 2011:365:474-475

1. Early Discontinuation of Individual Patients

The Investigator may remove a patient from study treatment at his/her discretion for any of the following reasons:

- Disease progression defined according to IMWG for progression of MM
- Unacceptable AE(s) or failure to tolerate the study treatment
- Any medically appropriate reason or significant protocol violation, in the opinion of the Investigator

Patients may discontinue study treatment for any reason. Patients who elect to discontinue study treatment should be encouraged to continue in the study so that follow-up information on disease progression and survival status may be obtained. However, patients may elect to withdraw consent and decline further participation in the study.

All patients will be followed until disease progression, withdrawal of consent, death, or loss to follow up.

1. **Planned Statistical Methods**

6.1 General Considerations

Tabulations will be produced for appropriate disposition, demographic, baseline, efficacy and safety parameters. For categorical variables, summary tabulations of the number and percentage of patients within each category (with a category for missing data) of the parameter will be presented, as well as two-sided 95% confidence intervals (CIs), unless otherwise stated. For continuous variables, the number of patients, mean, median, standard deviation, minimum, and maximum values will be presented. Time-to-event data will be summarized using Kaplan-Meier methodology using 25^th^, 50^th^ (median), and 75^th^ percentiles with associated 2-sided 95% CI, as well as percentage of censored observations.

6.2 Determination of Sample Size

*Dose Escalation Phase:*

The sample size for the Dose Escalation Phase of the study is based on the standard 3+3 dose escalation scheme typical in Phase-1 rising dose studies. Between 12 and 24 patients will be enrolled in the Dose Escalation Phase to determine the MTD for the two cohorts.

*Expansion Phase:*

Once the MTD has been determined for each Cohort, the SRC will use the results to inform their determination of the RP2D (by Arm) for the Expansion Phase.

The Expansion Phase analysis will be based on the modified intent-to-treat (mITT) population, where the first 10 patients in Arm 5 treated at the RP2D for that Arm are considered the first stage of the 2-Stage design.

The Expansion Phase is designed to test the following:

Test the null hypothesis that the true ORR is ≤ 0.30 against a 1-sided alternative that the true ORR is ≥ 0.6, requiring a sample size of 25 patients. After testing the combination in Arm 5 on the 10 patients in Stage 1, Arm 5 will be terminated if ≤ 3 patients respond. If Arm 5 proceeds to Stage 2, an additional 15 patients will be enrolled to include a total of 25 patients at the RP2D.

At the end of the study, if the total number of patients responding in an Arm is ≥ 45%, the treatment will be accepted as promising for further study for that treatment. For each Arm, this design achieves 80% power at a 1-sided 0.10 significance level.

6.3 Analysis Populations

*Dose Escalation Population (per Arm)*

The dose escalation population will consist of all patients in the Dose Escalation Phase who have either had a DLT prior to completion of one cycle of therapy, or who have completed a cycle of therapy.

*Modified Intent-to-Treat Population (per Arm)*

The modified intent-to-treat (mITT) population will consist of all patients who are assigned to study therapy and receive at least one dose of study treatment. This population will include patients who have discontinued therapy due to toxicity or disease progression and patients who have died from any cause, including those related to study drug or disease. This population will be used for primary analyses of efficacy and applies to the Expansion Phase only.

*Per Protocol Population (per Arm)*

The per-protocol (PP) population will consist of all patients who have received at least 2 cycles of study drug, are compliant with study assessments, have received at least 80% of their prescribed study medication, and have no major protocol violations that would compromise the assessment of efficacy. Major violations will be determined independently of knowledge of response to therapy, and prior to database lock and study analysis. This population will be used for supportive inferences concerning efficacy, however, if there are major differences between the results in this population and those obtained in the mITT population, this will be taken into consideration in the assessment of efficacy.

*Safety Population (All)*

The safety population will consist of all patients who have received ≥ 1 dose of study drug.

6.4 Demographics and Baseline Characteristics

Demographic characteristics will be summarized by cohort within treatment Arm (and by dose in the Escalation Phase) and will include gender, race, ethnicity (Hispanic/non-Hispanic origin), and age at time of consent. For gender, race, and ethnicity, the summary statistics will be the number and percentage of patients within each category. The categories for race will be those recorded in the database. For age at time of consent, the mean, median, minimum, maximum, and standard deviation will be provided for each group and the total sample.

Baseline characteristics include ECOG Performance Status, duration from initial diagnosis, response to previous therapy, types of prior therapy, and height/weight. Baseline data will be tabulated for the same categories as used for demographics, using summary statistics; no formal hypothesis testing of treatment Arm differences will be performed for either demographics or baseline characteristics.

Medical history and physical examination results at baseline will be tabulated by cohort.

6.5 Efficacy Analysis

*Primary Analysis: Overall Response Rate*

The analysis of ORR (primary endpoint) will be performed for each Arm separately, by calculating the point estimate of the percentage of patients in that Arm who have a response of sCR, CR, VGPR or PR, as assessed by IMWG criteria. To be consistent with the 2-stage design, a lower 1‑sided 90% CI will be presented for the ORR in each Arm; additionally, for descriptive purposes, a two-sided 95% CI will also be calculated for each Arm, using exact methods. The ORR for each Arm will be presented for the Stage 1 of the Expansion Phase and for both stages of the Expansion Phase combined (N=25 for Arm 5), consistent with the 2-stage design.

*Secondary Analyses*

Secondary analyses will be performed on the Expansion Phase (N ≈ 20 per Arm), within each Arm separately.

1. *Overall Response Rate (ORR) in patients with sFLC MM*: (MM that can be assessed by serum FLC, not M protein)
2. *Duration of Response*: DOR will be analyzed by Kaplan-Meier descriptive statistics for patients who have achieved overall response, with DOR calculated as the number of days from the date of the first evidence of objective response until progression. Patients who have not progressed at the time of analysis will be censored at the last available assessment date at which no evidence of disease was observed. Statistics will include the 25th, 50th (median), and 75th percentiles and associated 95% CIs, as well as the number and percentage of censored patients.
3. *Progression-free Survival*: PFS for patients in each Arm will be calculated from the date of start of study therapy to the date of progression based on IMWG criteria, or date of death due to any cause should progression not have occurred. Patients who drop out prior to study end will be censored at the last available assessment date at which no evidence of disease was observed. The analysis of PFS for patients in each Arm will be based on the Kaplan-Meier method for estimation of summary statistics, and include the 25th, 50th (median), and 75th percentiles and associated 95% CIs.
4. *Overall Survival*: OS for patients in each Arm will be calculated from the date of start of study therapy to the date of death due to any cause. This analysis will be performed in the same manner as the analysis of PFS.
5. *Clinical Benefit Rate*: The analysis of CBR will be performed for each study Arm separately, by calculating the point estimate of the percentage of patients in that Arm who have a response of sCR, CR, VGPR, PR or MR, as assessed by IMWG criteria. Analysis will be performed by calculation of the point estimate of CBR, as well as a two-sided 95% CI, using exact methods.
6. *Time to Progression*: TTP for patients in each Arm will be calculated from the date of start of study therapy to the date of first objective sign of progression.

6.6 Safety Analysis

Safety analyses will be performed using data available from all patients who receive ≥ 1 dose of study drug, including all patients who were in the Dose Escalation Phase or the Expansion Phase. Analyses will be presented by Arm and for all patients combined, and additionally by dose level, as appropriate. Note: analysis by study phase (Dose Escalation or Expansion) dose would be essentially superseded by analyses by dose level.

*Adverse Events*

AEs will be coded using the Medical Dictionary for Regulatory Activities (MedDRA) dictionary and displayed in tables and listings using System/Organ/Class (SOC) and Preferred Term.

Analyses of AEs will be performed for those events that are considered to be TEAEs, defined as any AE with onset or worsening of a pre-existing condition on or after the first administration of study medication through 30 days following last dose or any event considered drug-related by the Investigator through the end of the study. AEs with partial dates will be assessed using the available date information to determine if treatment-emergent; AEs with completely missing dates will be assumed to be treatment-emergent.

AEs will be summarized by patient incidence rates. In all tabulations, a patient may contribute only once to the count for a given AE preferred term.

The number and percentage of patients with TEAEs will be summarized, as well as the number and percentage of patients with TEAEs assessed by the Investigator as at least possibly related to treatment. The number and percentage of patients with any Grade ≥ 3 TEAE will be tabulated in the same manner. If a patient has repeated episodes of the same TEAE, the event with the highest severity and/or strongest causal relationship to study treatment will be used for purposes of tabulations.

Serious AEs (SAEs) will also be tabulated.

No formal hypothesis-testing analysis of AE incidence rates will be performed.

All AEs (treatment-emergent and post-treatment) will be listed in patient data listings.

Separate by-patient listings will be provided for the following: patient deaths, SAEs, and AEs leading to withdrawal.
